# Advancing hyperspectral imaging and machine learning tools toward clinical adoption in tissue diagnostics: A comprehensive review

**DOI:** 10.1063/5.0240444

**Published:** 2024-12-06

**Authors:** Chun-Liang Lai, Riya Karmakar, Arvind Mukundan, Ragul Kumar Natarajan, Song-Cun Lu, Cheng-Yi Wang, Hsiang-Chen Wang

**Affiliations:** 1 Division of Pulmonology and Critical Care, Department of Internal Medicine, Dalin Tzu Chi Hospital, Buddhist Tzu Chi Medical Foundation, No. 2, Minsheng Road, Dalin, Chiayi 62247, Taiwan; 2 Public School of Medicine, Tzu Chi University, 701 Zhongyang Rd., Sec. 3, Hualien 97004, Taiwan; 3 Department of Mechanical Engineering, National Chung Cheng University, 168, University Road, Min Hsiung, Chiayi City 62102, Taiwan; 4 Department of Biotechnology, Karpagam Academy of Higher Education, Salem - Kochi Hwy, Eachanari, Coimbatore, Tamil Nadu 641021, India; 5 Department of Gastroenterology, Kaohsiung Armed Forces General Hospital, 2, Zhongzheng 1st. Rd., Kaohsiung City 80284, Taiwan

## Abstract

Hyperspectral imaging (HSI) has become an evident transformative apparatus in medical diagnostics. The review aims to appraise the present advancement and challenges in HSI for medical applications. It features a variety of medical applications namely diagnosing diabetic retinopathy, neurodegenerative diseases like Parkinson's and Alzheimer's, which illustrates its effectiveness in early diagnosis, early caries detection in periodontal disease, and dermatology by detecting skin cancer. Regardless of these advances, the challenges exist within every aspect that limits its broader clinical adoption. It has various constraints including difficulties with technology related to the complexity of the HSI system and needing specialist training, which may act as a drawback to its clinical settings. This article pertains to potential challenges expressed in medical applications and probable solutions to overcome these constraints. Successful companies that perform advanced solutions with HSI in terms of medical applications are being emphasized in this study to signal the high level of interest in medical diagnosis for systems to incorporate machine learning ML and artificial intelligence AI to foster precision diagnosis and standardized clinical workflow. This advancement signifies progressive possibilities of HSI in real-time clinical assessments. In conclusion despite HSI has been presented as a significant advanced medical imaging tool, addressing its limitations and probable solutions is for broader clinical adoption.

## I. INTRODUCTION

**FIG. 1.**
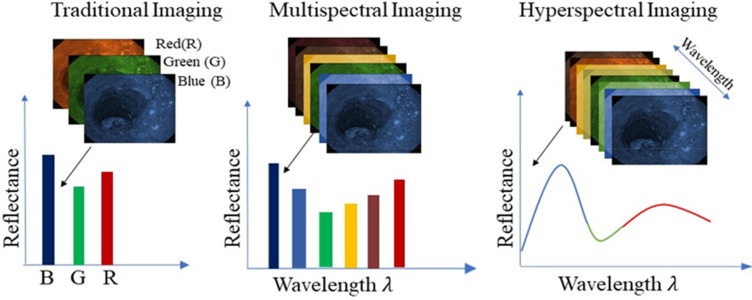
Comparison of imaging modalities used in tissue diagnostics, illustrating the differences between traditional RGB imaging, multispectral imaging, and HSI. Traditional imaging captures limited spectral information across the RGB channels, providing a coarse representation of reflectance across broad wavelength bands. Multispectral imaging extends this by capturing several discrete spectral bands, enabling improved tissue characterization. HSI further refines this approach by capturing continuous spectral information across many wavelengths, resulting in detailed reflectance spectra for each pixel. This enhanced spectral resolution allows HSI to detect subtle biochemical and structural variations within tissues, facilitating early disease diagnosis and more accurate tissue differentiation.

In recent decades, researchers have emphasized advancing noninvasive optical techniques for diagnosing optical properties of pathologic tissues.^1^ One of the noninvasive techniques applied in clinical research is hyperspectral imaging (HSI); it involves aggregating hundreds of band data, with each band wrapping a straight and adjacent segment of the electromagnetic spectrum.^2^ HSI estimates the two-dimensional (2D) structural data with one-dimensional (1D) spectral information with the appearance of a three-dimensional (3D) dataset called a “hypercube,” which provides a classification of the target sample beyond what traditional color imaging methods can provide.^3^ HSI captures accurate spectral information throughout many bands as compared to RGB cameras, allowing it to examine subtle differences in pathology as shown in Fig. 1. Thus, HSI is good at capturing nuanced spectral features that RGB imaging might miss.^4^

In 1997, HSI gained attention in clinical research when Freeman *et al.* used HSI to enhance a surgeon's ability to identify unpredictable issues during clinical procedures by providing extended vision beyond what the naked eye can perceive.^5^ Then, in the year 1998, Mooradian *et al.* developed patented equipment to detect cancer locations and the degree of cancer diffusion to identify cancerous tissues.^6^ In the same year, more researchers began inceptive investigations into cervical cancer detection and diagnosis.^7^ By 2005, hemoglobin saturation in tumor microvasculature and hypoxia was monitored using HSI.^8^ The utilization of HSI for discerning changes in oxygenation saturation levels marked a substantial progress in noninvasive wound assessment and metabolic correlation.^9^ Then, in 2006, HSI enabled *in vivo* observation of cutaneous edema by allowing to analyze the evident concentration of water at each pixel with oxyhemoglobin and deoxyhemoglobin to build concentration maps for these molecules.^10^ In the year 2012, newly developed HSI classification techniques guaranteed the potential for noninvasive prostate cancer detection, providing surgeons with the ability to evaluate tissue without the need for physical biopsies.^11^ Brain tumors were detected in pathological slides using HSI in the year 2018 and the results demonstrate that HSI is an effective method for automatically identifying high-grade tumors in pathological slides.^12^ By 2022, HSI was an evolving medical imaging tool that would extend valuable spatial and spectral insights into tissue samples, enabling potential applications in deep learning in medical diagnosis.^13^

Regardless of HSI being established as a highly advanced tool for clinical imaging over traditional imaging systems, the predominant consumption of HSI in clinical settings remains uncertain. This review mainly focuses on bridging the gap between HSI towards clinical adoption by focusing on its fundamental concepts. This study highlights the transformative potential of integrating ML and AI with HSI to enhance *in vivo* tissue diagnostics, notably in cancer applications. ML and AI algorithms can effectively process HSI's complex datasets, identifying patterns and biomarkers that are often challenging to detect through traditional imaging. This capability supports earlier, more precise disease detection while also enabling automation, which minimizes the need for specialized training, increasing HSI's practicality in clinical settings. Additionally, AI-driven HSI systems offer noninvasive, real-time diagnostic insights, streamlining workflows and ultimately improving patient outcomes by providing rapid, personalized diagnostic information. To assess advancements in HSI, this review applied rigorous inclusion and exclusion criteria to select relevant research. The inclusion criteria required studies to^1^ present definitive numerical results, including metrics like dataset size, sensitivity, accuracy, precision, and area under the curve (AUC).^2^ Further, the study concentrates on HSI applications,^3^ be published within the past 6 years, employ prospective or retrospective study designs, and^5^ be written in English. Studies falling under the exclusion criteria were omitted, including those with insufficient data, narrative reviews, systematic reviews, meta-analyses, comments, proceedings, study protocols, and conference papers. This selection approach ensures a focus on recent, data-driven studies with measurable outcomes, thereby offering a comprehensive and current overview of HSI developments in medical diagnostics. In this review, cancer diagnostics are discussed within relevant tissue-specific sections, such as skin and ocular tissues, to provide a clear and organized framework and biomolecular diagnostics and HSI enhancements involving nanomaterial contrasts are excluded.^4^

## II. LITERATURE REVIEW

In early research studies, HSI classification primarily relied on imposing prior knowledge to gather both spatial and spectral data for the sorting process.^14^ Spatial scanning HSI employs dispersive optical elements like prisms and gratings to calculate reflected light intensity across wavelengths. The two primary approaches for spatial scanning are “whiskbroom” and “pushbroom.”^15^ The whiskbroom approach involves using a pinhole to scan the image point by point systematically, necessitating scanning across both spatial dimensions of the image.^16^ In the year 2017, Wang *et al.* used whiskbroom mode for creating HSI cubes of acute lymphoblastic leukemia.^17^ Bassler *et al.* used whiskbroom imaging for differentiating types of tissue in head and neck squamous cell carcinoma (HNSCC) by generating unique electroluminescence spectroscopy (ELS) spectra for epithelial, glandular, and adipose tissues, improving diagnostic accuracy.^18^ Whiskbroom scanning employed by Taylor *et al.* allowed to acquire high-resolution pictures from the burned skin of porcine and it classified the difference between the area that was burned and unburned with a 20 dB SNR difference when functioning at 500 GHz with 125 GHz bandwidth.^19^ The pushbroom approach applies a slit aperture for line-by-line scanning of the image, it is only essential to scan down one spatial dimension of the image. Hence, this method is quicker and more efficient.^20^ Le *et al.* employed the pushbroom methodology in diabetic retinopathy research where microscopic pushbroom HSI allowed observation of retina segments of diabetic, normal, and treated diabetic rats. The spectral angle mapper (SAM) algorithm allowed detection of spectral differences, indicating the potential for identifying biochemical changes.^21^ In 2015 Lim *et al.* used pushbroom for biomedical imaging, particularly for analyzing biological tissues such as chicken breast tissue and fluorescent targets integrating a video camera enabling efficient region selection, enhancing its potential in endoscopic imaging.^22^ Muhle *et al.* highlight that the pushbroom camera exhibits a correlation with ground truth data in organ transplantation imaging, owing to their high spectral resolution and ability to precisely detect tissue properties and abnormalities such as cysts.^23^ Spectral scanning is dealt with a liquid crystal tunable filter (LCTF), which is either placed in front of the illumination source or the image sensor; these will capture a streak of images in 2D of the target at distinct wavelengths. After aligning, the captured image will create a hyperspectral cube.^24^ Bartczak *et al.* employed an LCTF-based spectral imaging system for collecting data on-site; it is suitable for other medical applications like tumor resections, epilepsy surgeries, and cholesteatoma treatment.^25^ Mun *et al.* used LCTF for tumor detection in biomedical research by utilizing an endoscopic hyperspectral imaging (eHSI) system.^26^ The snapshot method captures the pair of spatial and spectral information simultaneously in a single measurement without scanning.^27^ Kester *et al.* developed a concurrent snapshot HSI endoscope that is used *in vivo* for a high speed by offering an (x, y, *λ*) data cube of 350 × 350 × 48 at 5.2 frames per second. It provides 10 to 100 spectral channels for every image pixel and serves to be a powerful tool for detecting cancer at an early stage by allowing detailed spectral information.^28^ Hedde *et al.* stated that vigorous phasor-based hyperspectral snapshot microscopy uses a sine/cosine boundary filter to increase imaging speed and efficiency and this method is suitable for live tissue studies since it allows rapid and high-resolution HSI.^29^ Lim *et al.* discovered a four-dimensional (4D) video endoscope with snapshot-hyperspectral, which captures images across 400–1000 nm for *in vivo* biomedical imaging.^30^

**FIG. 2.**
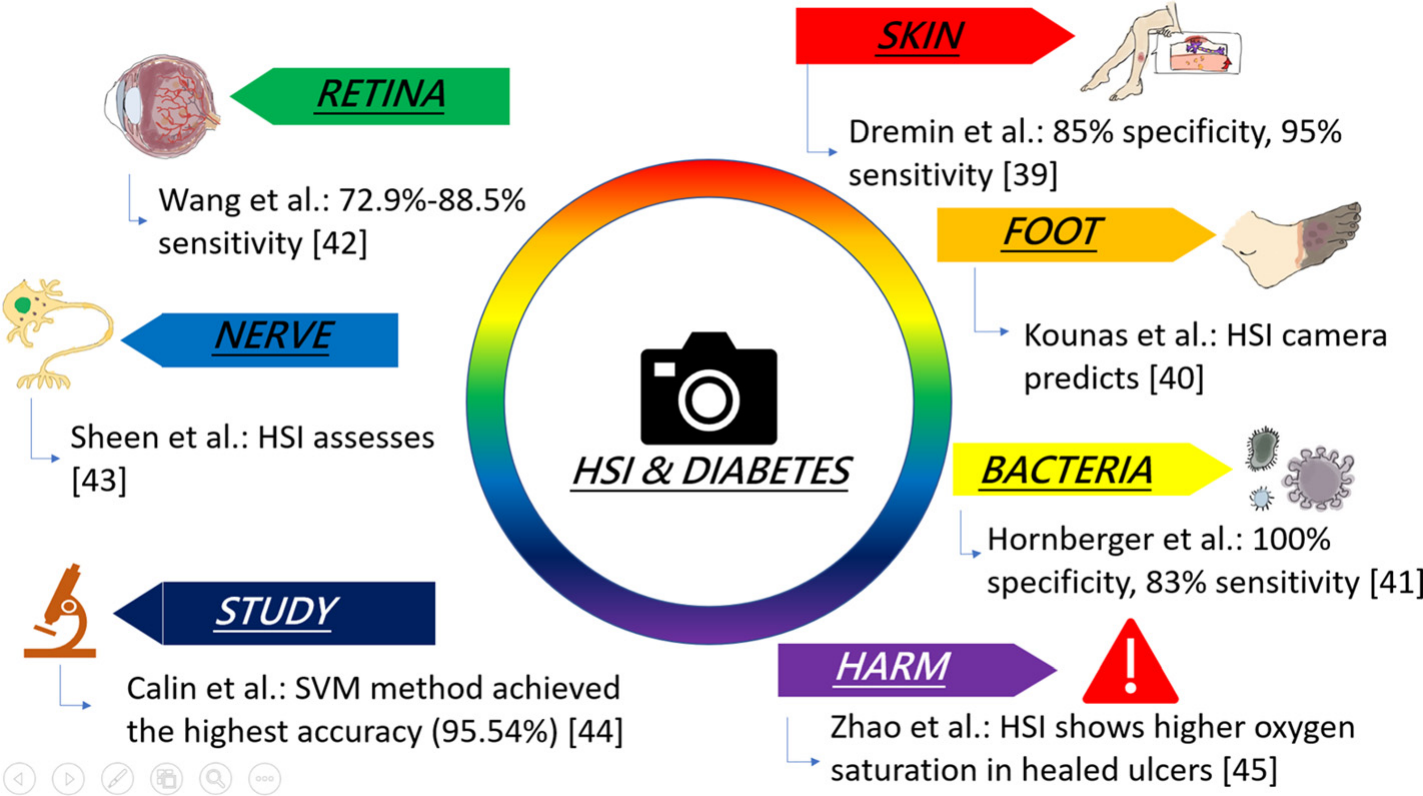
Overview of HSI studies in assessing diabetic complications.

HSI is a highly developed visual imagery technique that augments the principles established by traditional white light imaging (WLI) and narrow-band imaging (NBI).^31^ NBI is considered superior to WLI due to its ability to enhance mucosal and vascular detail.^32^ Cancer detection relies heavily on NBI because it enhances the surface characteristics of the mucosa.^33^ NBI is a clinical imaging method that uses a special filter only to allow specific wavelengths to pass through.^34^ These wavelengths possess the capacity to boost contrast, and as a result, the characteristics of the mucosal layer are highlighted.^35^ NBI operates on the principle of light penetration properties. Shorter wavelengths (blue spectrum) accentuate capillaries in the superficial mucosa by primarily absorbing hemoglobin at 415 nm. In contrast, longer wavelengths (green spectrum, 540 nm) penetrate deeper into the mucosa.^36^ This improves mucosal surface clarity by enhancing the variation between the mucosa and superficial vessels, which appear brown.^37^ NBI enables better evaluation of vascular and mucosal patterns linked to gastrointestinal lesions and cancers.^38^

## III. CURRENT STATE OF HSI IN MEDICINE

### A. Diabetes

Dermin *et al.* proposed a method that is used to diagnose skin complications at an early stage of diabetes mellitus by performing an artificial neural network (ANN) with a wavelength resolution of 6–10 nm offering a frequency range from 510–900 nm and achieving specificity 85%, sensitivity 95% with participation of 20 patients as shown in Fig. 2.^39^ A study by Kounas *et al.* states that a portable HSI camera can predict foot ulcer rehabilitation with a measured oxyhemoglobin specificity of 70% and a sensitivity of 85% on the first visit, and for the second visit it is estimated to be 85% for specificity and an 85% for sensitivity by examining 27 patients with foot ulcer.^40^ Hornberger *et al.* examined the bacterial colonization in chronic non-healing wounds of diabetic foot ulcers using HSI for 42 patients by employing the technique called principal component analysis (PCA) results with a specificity of 100% and sensitivity of 83%, the fluorescence portraits obtained at the wavelength emission of 510–745 nm with a spectral resolution of 5 nm.^41^

In 2023, Wang *et* *al.*,^42^ used HSI for inspecting the fundus images to evaluate the severity of diabetic retinopathy (DR) from 91 patients with a wavelength of 380–780 nm; the study reported the sensitivity for detecting arteries as 82.4% in normal stage, 77.5% in background DR (BDR), 78.1% in pre-proliferative DR (PPDR), and 72.9% in proliferative DR (PDR). For the veins, it was stated to be 88.5% in the normal stage, 85.4% in BDR, 81.4% in PPDR, and 75.1% in PDR.^42^ Sheen *et al.,* employed HSI for the investigation of diabetic neuropathy (DN) in a study involving 120 patients with type two diabetes mellitus; DN assessment incorporated standard methods such as the Michigan neuropathy screening instrument (MNSI), pain evaluations, and sudomotor function via electrochemical skin conductance (ESC).^43^ A study by Calin *et al.* examined the most accurate technique for differentiating between normal and pathological tissues in hyperspectral images of diabetic feet; the study compared four ML techniques: SAM, minimum distance technique (MD), support vector machine (SVM), and spectral information divergence (SID). SVM was found to be the most accurate of all the above-mentioned techniques with an overall accuracy of 95.54%.^44^ Zhao *et al.* reconstructed HSI images from optical data related to diabetic foot ulcers, which indicate healed ulcers had an average oxygen saturation ranging from 54% to 64% and non-healed ulcers showed oxygen saturation levels between 32% and 48%.^45^

### B. Neurodegenerative disease

**FIG. 3.**
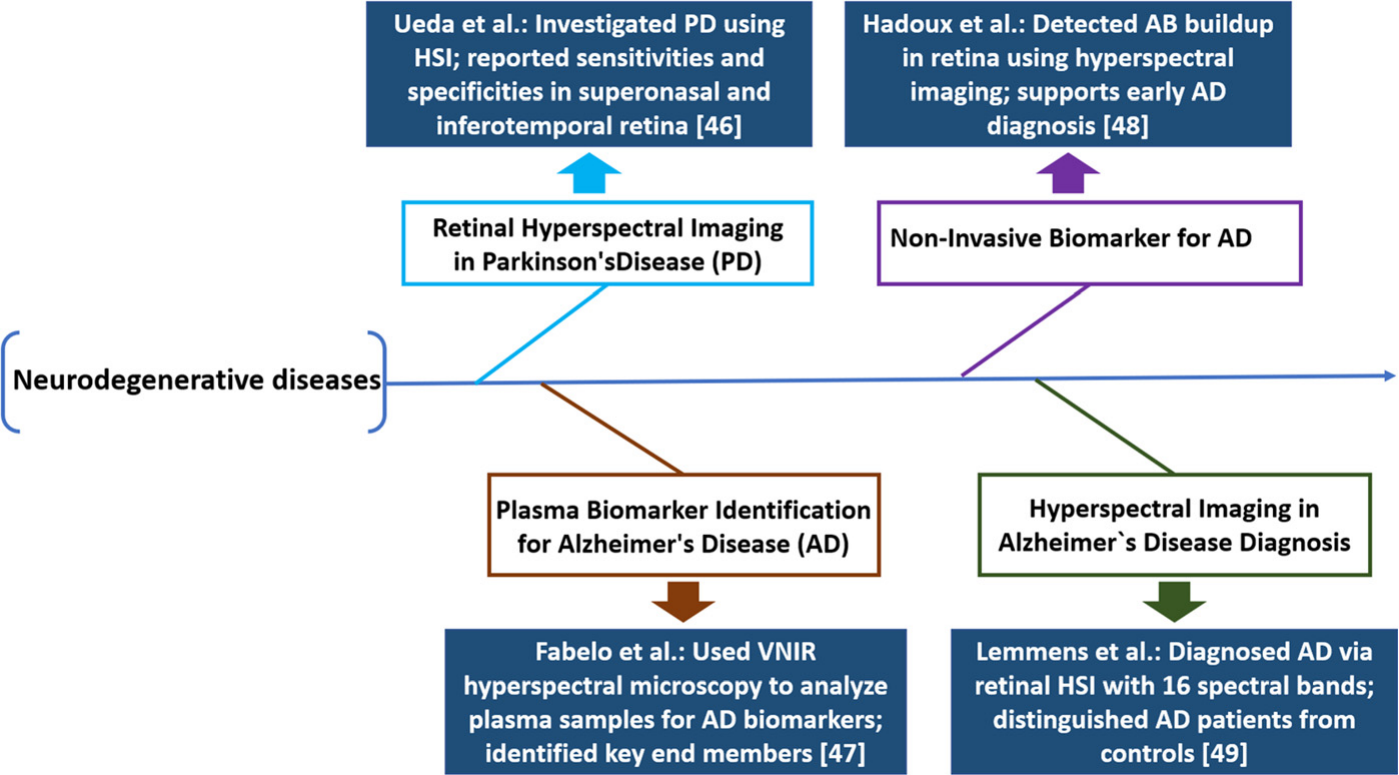
Summary of HSI research in neurodegenerative disease assessment.

The potential of retinal HSI related to neurodegenerative disorders has been studied by Ueda *et al.*, particularly Parkinson’s disease (PD). Their study involved 20 PD patients and analyzed the superonasal and inferotemporal retina using a wavelength range of 463–598 nm. The result findings indicated a sensitivity of 60%, a specificity of 55%, and an accuracy of 57.5% in the super nasal retina, while the inferotemporal retina exhibited a sensitivity of 60%, a specificity of 50%, and an accuracy of 55%.^46^ An innovative approach has been introduced by Fabelo *et al.* using spectral unmixing to reorganize biomarkers in plasma samples through visible and near-infrared (VNIR), hyperspectral microscopy (HSM). Plasma samples from 10 patients are examined by using hyperspectral retinal cameras with a wavelength range of 450–900 nm at a frequency resolution of 2.8 nm. Statistical analysis implies that HSM of plasma samples is a cost-effective method for early Alzheimer's disease (AD) diagnosis.^47^ A study by Hadoux *et al.* demonstrates that retinal HSI can serve as a non-invasive biomarker for AD by detecting amyloid beta (A*β*) buildup. Hyperspectral cameras capture the spectral data in the VNIR spectrum of 400 to 1000 nm alongside a spectral resolution of 2.8 nm, the results demonstrate that this method could precisely predict brain Aβ load, aiding in the diagnosis of AD.^48^ By studying retinal images from 17 AD patients, Lemmens *et al.* demonstrated that an HSI snapshot camera captures 16 spectral bands from 460 to 620 nm, effectively distinguishing AD patients from controls and highlighting the benefits of combining multiple retinal imaging techniques as illustrated in Fig. 3.^49^

### C. Periodontal disease

**FIG. 4.**
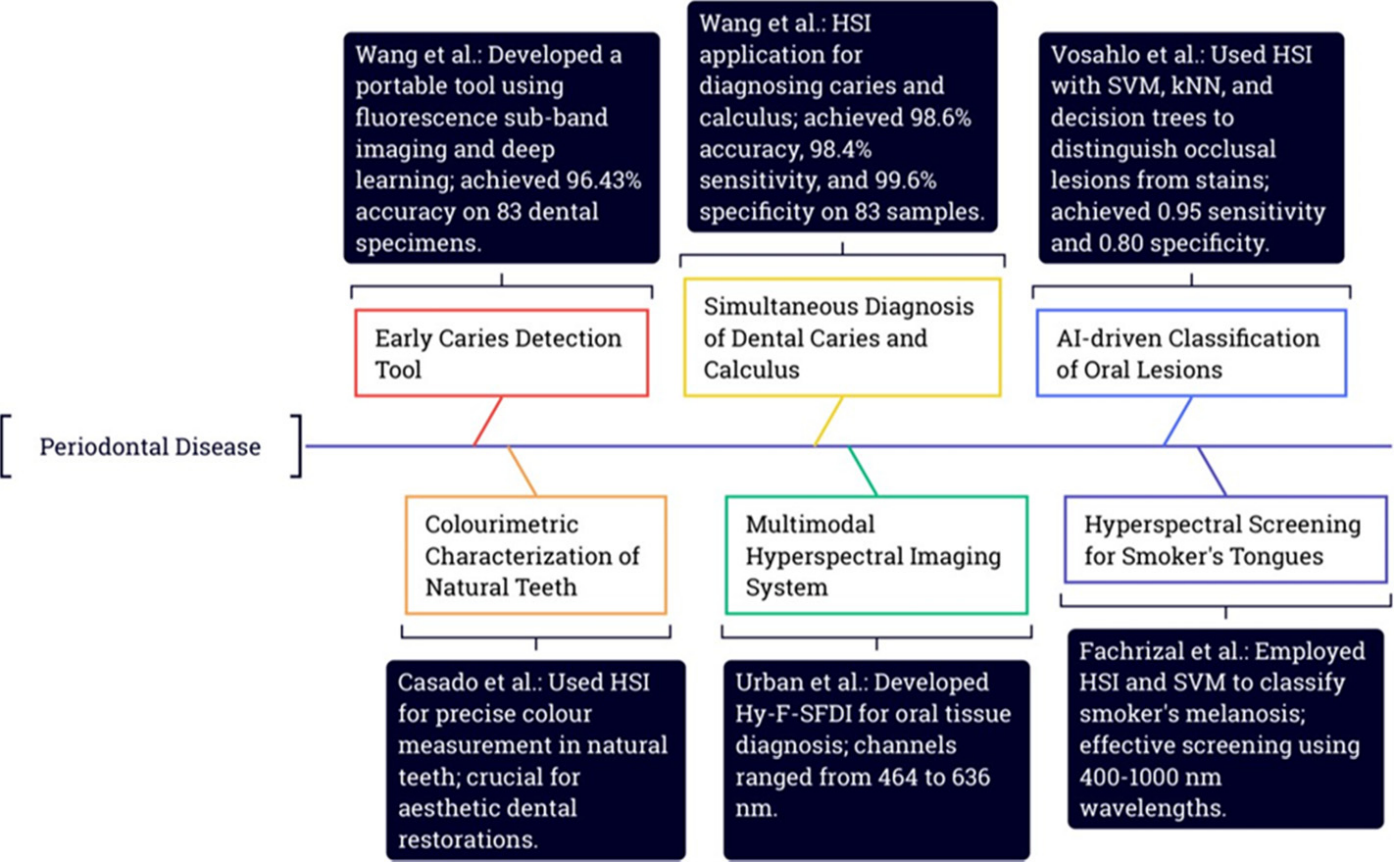
Exploring hyperspectral imaging in the evaluation of periodontal conditions.

A portable and automated tool for early caries detection using fluorescence subband imaging and learning was developed by Wang *et al.*, which captured six-channel fluorescence images (470–780 nm) and was tested on 83 dental specimens, resulting in an accuracy of 96.43% by utilizing a 2D–3D hybrid convolutional neural network (CNN) with attention mechanisms for classification.^50^ A mobile HSI camera with a spectral range of 400–1000 nm wavelength and a spectral precision of about 3 nm was utilized by Casado *et al.* for precise colorimetric portrayal of *in vivo* natural teeth.^51^ A study by Wang *et al.* demonstrated a diagnostic model for dental caries and calculus that had an accuracy of 98.6%, sensitivity of 98.4%, and specificity of 99.6% in characterizing four different stages of caries and calculus with 83 dental samples.^52^ A multisensory HSI system called hyperspectral-fluorescence-spatial frequency domain imaging (Hy-F-SFDI) was developed by Urban *et al.* for the diagnosis of oral tissues with the HSI device channels spanning a range from 464 to 636 nm.^53^ A study by Vosahlo *et al.* distinguishes early occlusal lesions from stains by employing HSI with AI-driven classification algorithms like SVM, kNN algorithm, and decision tree, whence the kNN algorithm demonstrated notable performance with a sensitivity of 95% and specificity of 80%, constructively classifying differences among intact stained enamel and stained lesions within the wavelength range of 505–1000 nm.^54^ Fachrizal *et al.* utilized hyperspectral screening ranging from 400 to 1000 nm and a custom image processing software to characterize smoker and nonsmoker tongues. The reflectance values are interrupted with PCA and SVM classifier, and the system accurately classifies smoker's melanosis with notable accuracy as shown in Fig. 4.^55^

### D. Dermatology

The detection of cutaneous malignant melanoma (cMM) in Caucasian skin types studied by Christensen *et al.*, demonstrated an HSI device with an optical range from 400 to 800 nm with a resolution of 2.4 nm. This device was evaluated for its efficacy in differentiating cMM from benign pigmented skin lesions (PSL) among 186 patients with 202 PSLs. The results exhibit a sensitivity 96.7% and specificity 42.1%, with high sensitivity highlighting the potential of this promising tool for the triage of pigmented skin lesions.^56^ A study by Kye *et al.*, involving 23 patients with atopic dermatitis and using image wavelengths from 470 to 900 nm, demonstrated that HSI magnifies the accuracy and reliability of erythema severity classification. It serves to be a promising tool for application in skin pigmentation studies.^57^ A noninvasive technique for assessing atopic dermatitis (AD) using HSI by Kim *et al.* demonstrated that ResNet50 achieved the highest classification accuracy of 81%. The study involved 25 patients with AD and HSI camera SNAPSCAN VNIR covering wavelengths from 470 to 900 nm with a resolution of 2.8 nm.^58^

The study of HSI for examining the skin carcinoma margins preoperatively by Parasca *et al.* demonstrated the capture of hyperspectral images of six basal cell carcinomas (BCCs) and five squamous cell carcinomas (SCCs). It emphasizes HSI's ability to objectively delineate tumor margins by employing a spectral span of 400–800 nm and a spectral precision of 1.97 nm.^59^ Calin *et al.* demonstrated that HSI combined with unsupervised anomaly detectors can profoundly distinguish BCCs from normal skin. A 64-year-old female patient with lower lip BCC was analyzed using a visible/near-infrared HSI system with a spectral range of 400–800 nm, a wavelength resolution of 1.97 nm, and an area under the curve value of 0.8607 and 0.7074. The result illustrates the potential of HSI for automated BCC detection.^60^

Saknite *et al.* conducted a study with 51 lesions from 15 patients diagnosed with chronic graft-vs-host disease using a wavelength range from 450 to 850 nm and a spectral resolution of 3.3 nm. This study demonstrates that HSI effectively identified active erythema and post-inflammatory hyperpigmentation.^61^ Räsänen *et al.* employed HSI to distinguish pigmented BCC from melanocytic tumors with a sensitivity of 100% and specificity of 90% in a pilot consisting of 26 lesions by utilizing 3D, 2D, and 1D CNN across both spatial and spectral domains in the bandwidth ranging from 450 to 850 nm.^62^ Paoli *et al.* examined 285 patients, used deep neural network and pixel-wise classification methods, and demonstrated that HSI has the potential to differentiate melanoma from benign lesions with a sensitivity and specificity of 95% and 92%.^63^

Nowak *et al.* used HSI to evaluate the contrast between the reflective index of acne skin prior to and after intense pulsed light (IPL) therapy with healthy skin. The study included 27 acne patients and 20 healthy controls, with wavelength ranging from 397 to 1004 nm and a spectral definition of 3 nm.^64^ In a study by Nowak *et al.*, the authors utilize HSI for evaluating microcirculation in acne lesions in contrast to healthy skin using laser speckle contrast analysis (LASCA) for semi-quantitative blood perfusion measurement and analysis of image methods, and the results indicated that blood perfusion in acne lesions was 117% elevated than in healthy skin in the range 400–1000 nm.^65^ Non-invasive detection and classification systems of pigmented skin lesions (PSLs) were investigated by Leon *et al.*, capturing 125 spectral bands within the wavelength range of 450–950 nm from the 76 PSL images of 61 patients. The system secured a sensitivity 87.5% and a specificity 100%, indicating its effectiveness in detecting PSLs.^66^

HSI and spectral phasor analysis were employed by Schuty *et al.* to differentiate melanoma from intradermal nevi by examining tissue autofluorescence patterns in the 423–723 nm range.^67^ Huang *et al.* used HSI for the detection and classification of skin cancer thus including BCC, SCC, and seborrheic keratosis (SK). They trained and assessed a YOLO V5 model using precision, recall, specificity, and accuracy to effectively sort and identify these skin conditions. The model operated within the wavelength range of 380–780 nm and with a spectral resolution of 1 nm.^68^ Salvia *et al.* created a deep learning pipeline using HSI with a wavelength in the range 450–950 nm and spectral precision of 8 nm. ResNet 3D achieved 87% sensitivity and 88% specificity and was reported to have the best performance,^69^ as summarized in Table I.

**TABLE I.** Clinical applications of hyperspectral imaging in dermatology.

Study	HIS application	Wavelength range	Spectral resolution	Patient s	Result	Methods
Christense n *et al.*	Detection of cutaneous malignant melanoma (cMM) in Caucasian skin types	400–800 nm	2.4 nm	186	Sensitivity: 96.7%, specificity: 42.1%	Differentiating cMM from benign pigmented skin lesions
Kye *et al.*	Classification of erythema severity in atopic dermatitis	470–900 nm	N/A	23	N/A	A promising tool for skin pigmentation studies
Kim *et al.*	Classification of atopic dermatitis	470–900 nm	2.8 nm	25	Accuracy: 81%	ResNet50 model
Parasca *et al.*	Examining skin carcinoma margins preoperatively	400–800 nm	1.97 nm	11	N/A	Objectively delineating tumor margins
Calin *et al.*	Automated detection of BCC from normal skin	400–800 nm	1.97 nm	1	AUC: 86.07%, 70.74%	Unsupervised anomaly detectors
Saknite *et al.*	Identifying active erythema and post-inflammatory hyperpigmentation	450–850 nm	3.3 nm	15	N/A	Chronic graft- vs-host disease
Räsänen *et al.*	Distinguishing pigmented BCC from melanocytic tumors	450–850 nm	N/A	26	Sensitivity: 100%, specificity: 90%	3D, 2D, and 1D CNN
Paoli *et al.*	Differentiating melanoma from benign lesions	N/A	N/A	285	Sensitivity: 95%, specificity: 92%	Deep neural network and pixel-wise classification
Nowak *et al.*^1^	Evaluating reflective index of acne skin pre- and post-IPL therapy	397–1004 nm	3 nm	47	N/A	Contrast with healthy skin
Nowak *et al.*^2^	Evaluating microcirculation in acne lesions	400–1000 nm	N/A	47	Blood perfusion: 117% elevated	Laser speckle contrast analysis (LASCA)
Leon *et al.*	Detection and classification of pigmented skin lesions (PSLs)	450–950 nm	N/A	61	Sensitivity: 87.5%, specificity: 100%	125 spectral bands
Schuty *et al.*	Differentiating melanoma from intradermal nevi	423–723 nm	N/A	N/A	N/A	Tissue autofluorescence patterns
Huang *et al.*	Detection and sorting of skin cancer (BCC, SCC, SK)	380–780 nm	1 nm	N/A	N/A	YOLO V5 model
Salvia *et al.*	Detection of skin cancer	450–950 nm	8 nm	N/A	Sensitivity: 87%, specificity: 88%	ResNet 3D model

### E. Cancer

The clinical use of commercially available HSI systems (TIVITA^®^) has been assessed by Schimunek *et al.* for the detection of cervical intraepithelial neoplasia (CIN) stages 1–3 in a trial involving 41 patients at wavelength ranging from 500 to 995 nm with a resolution of 5 nm. The findings show the potential of the HSI method for practical diagnosis of CIN.^70^ A novel HSI platform introduced by Youssef *et al.* integrated fuzzy c-means clustering for machine-driven breast cancer detection in six different patients with a wavelength ranging from 390 to 980 nm and a spectral resolution of 5 nm and reported a high sensitivity of 96.83%, a specificity of 93.39%, and an accuracy of 95.12%.^71^ The study by Ortega *et al.* demonstrated that HSI provides exhaustive information about tissue composition and morphology. They integrated HSI with deep learning to differentiate glioblastoma from non-tumor tissues. They performed utilizing the HSI microscope with a bandwidth of 400 to 1000 nm and a resolution of 2.8 nm by relaying on the CNN method. The results showed a sensitivity of 88% with a specificity of 77%, with improvements of over 7% and 8% in specificity compared to traditional RGB images.^72^

Mubarak *et al.* simulated a polarized hyperspectral imaging (PHSI) system for hematoxylin and eosin (H&E) stained pathological slides and developed a dataset for deep learning classification utilizing CNN covering a spectrum span of 470–900 nm with a spectral sharpness of 2.8 nm for a PHSI microscope and captured hypercubes from 56 patients. The results from the CNN model showed an accuracy of 96.3%, a sensitivity of 98.6%, and a specificity of 94%.^73^ The application of HSI in thyroid pathology was evaluated by Baffa *et al.*, who progressed with a significant result in k-fold cross-validation with a high accuracy of 93.66%, a sensitivity of 93.47%, and a specificity of 96.93%, using wavelengths ranging from 400 to 700 cm^−1^.^74^ In the detection and classification of gastric precancerous lesions Wang *et al.*, used a novel RBG and hyperspectral dual-modality imaging feature fusion network (DuFF-Net) for covering the wavelength range from 450 to 700 nm. The approach resulted in a substantial improvement in screening accuracy of 96.15%.^75^

A study by Muniz *et al.*, explored how HSI through micro Fourier-transform infrared spectroscopy (FTIR) absorbance spectroscopy, could enhance the characterization of colon tissues. They achieved 99% accuracy with deep neural networks and 96% with linear SVM through k-fold cross-validation from 55 patients across spectral data collected from 750 to 4000 cm^−1^. The finding indicates the potential of HSI to increase diagnostic capabilities in colon pathology.^76^ An analysis by Filho *et al.* investigated how HSI can help in diagnosing pancreatic cancer by utilizing partial least squares discriminant analysis (PLS-DA) to segregate carcinogenic tissues acquired using the SisuCHEMA workstation, which is optimized for short wave infrared (SWIR) spectrum from 1000 to 2500 nm.^77^ An innovative method using HSI for detecting and classifying oral cancer in the maxillofacial area has been illustrated by Jeyaraj *et al.* by utilizing HSI precise wavelength (500–950 nm) resolution, achieving a remarkable accuracy of 94.75% through classifier fusion.^78^

Tsai *et al.* in the year 2021 carried out analysis to enhance the diagnosis of esophageal cancer stage using HSI and detect lesions through a deep learning approach and achieved impressively high accuracy of 83% for white light and 88% for NBI. The improved significant difference was observed comparing the traditional RGB-based method and NBI, which achieved accuracies of 86% and 91%, respectively.^79^ Collins *et al.*, enhanced HSI for the detection of colorectal and esophagogastric cancer within a spectral range of 500–1000 nm. They trained several ML models including 3DCNN, SVM with radial basis function kernel, and multi-layer perceptrons (MLP). To improve the detection accuracy. The 3DCNN model obtained the highest performance with an area under the receiver operating characteristic curve (ROC-AUC) of 0.93 for both cancer types.^80^ A study by Collins *et al.* explored improving the intraoperative detection of colorectal cancer using HSI by developing CNN to interpret data from 34 patients by accurately differentiating cancerous tissue from normal tissue. The CNN achieved a high sensitivity of 87%, a specificity of 90%, and an impressive ROC-AUC score of 0.95.^81^

An early breast cancer detection protocol using HSI has been developed by Sharkawy *et al.*, employing a polychromatic light source spanning 400–900 nm. The study utilized a cross-correlation algorithm, moving average filter, and K-means clustering for image analysis where HSI demonstrated high accuracy, specifically with reflected images at 600 nm and transmitted images at 750 nm.^82^ Utilizing HSI to improve intraoperative tissue detection in neurosurgery was explored by Puustinen *et al.*, focusing on glioma surgeries, Using a tunable Fabry-Perot interferometer within the 500–900 nm wavelength range. The study enhances a deep learning approach and achieves an accuracy of 80% in multi-tissue classification.^83^ Halicek *et al*., examined HSI for tumor detection in *ex-vivo* thyroid and salivary gland, the evaluation involved 82 patients. The study used a spectral range of 450–900 nm with a 5 nm spectral resolution and acquired a xenon light source along with an LCTF. A CNN model was utilized for accurate sorting of thyroid tissue into tumor categories, achieving an AUC score of 0.90 for tumor and a patch-based approach for sorting normal tissue. The salivary gland tumors achieved an AUC score of 0.92 as indicated in Table II.^84^

**TABLE II.** Cancer diagnostics enhanced by HSI.

Study	HSI application	Wavelength range	Patients	Accuracy (%)	Sensitivity (%)	Specificity (%)	Methods
Youssef *et al.*	Breast cancer detection	390–980 nm	6	95.12	96.83	93.39	Fuzzy c-means clustering
Munarak *et al.*	Classification of H&E-stained pathological slides	470–900 nm	56	96.3	98.6	94	Polarized HIS microscope, CNN model
Baffa *et al.*	Thyroid pathology	400–700 cm^−1^	N/A	93.66	93.47	96.93	k-fold cross-validation
Wang *et al.*	Detection of gastric precancerous lesions	450–700 nm	N/A	96.15	N/A	N/A	DuFF-Net, dual-modality imaging
Muniz *et al.*	Characterization of colon tissues	750–4000 cm^−1^	55	99	N/A	N/A	Micro-FTIR absorbance spectroscopy, deep neural networks
Filho *et al.*	Diagnosis of pancreatic cancer	1000–2500 nm	N/A	N/A	N/A	N/A	PLS-DA, SisuCHEM A workstation optimized for SWIR
Jeyaraj *et al.*	Detection and classification of oral cancer	500–950 nm	N/A	94.75	N/A	N/A	Classifier fusion
Tsai *et al.*	Diagnosis of esophageal cancer	N/A	N/A	88	N/A	N/A	Deep learning approach
Collins *et al.*^1^	Detection of colorectal and esophagogastric cancer	500–1000 nm	N/A	N/A	N/A	N/A	3DCNN, SVM with RBF kernel, MLP
Collins *et al.*^2^	Intraoperative detection of colorectal cancer	N/A	34	N/A	87	90	CNN model
Sharkawy *et al.*	Early breast cancer detection	400–900 nm	N/A	N/A	N/A	N/A	Polychroma tic light source, cross correlation algorithm
Puustinen *et al.*	Intraoperative tissue detection in neurosurgery	500–900 nm	N/A	80	N/A	N/A	Tunable Fabry–Perot interferometer, deep learning
Halicek *et al.*	Detection of tumors in *ex vivo* thyroid and salivary gland	450–900 nm	82	N/A	N/A	N/A	CNN model, liquid crystal tunable filter (LCTF)
Sharkawy *et al.*	Early breast cancer detection	400–900 nm	N/A	N/A	N/A	N/A	Polychroma tic light source, cross-correlation algorithm
Puustinen *et al.*	Intraoperative tissue detection in neurosurgery	500–900 nm	N/A	80	N/A	N/A	Tunable Fabry–Perot interferometer, deep learning

### F. Ophthalmology

Sharkawy *et al.*, demonstrated a noninvasive characterization of human eye vasculature using HIS. By employing the k-means clustering method, the study used a spectrum spanning 400–1000 nm with a 1.3 nm resolution. The study identified a wavelength of 470 nm for arteries and 750 nm for veins, 10 volunteers' data were analyzed for the study. The results demonstrated the differences between arteries and veins with the potential of HSI for mapping varicose veins.^85^ Tran *et al.* developed a novel imaging system where high-resolution RGB camera is combined with a snapshot hyperspectral camera to capture intricate HSI images of the retina with spatial resolution ranging from 0.2 to 0.1 mm/pixel.^86^

**FIG. 5.**
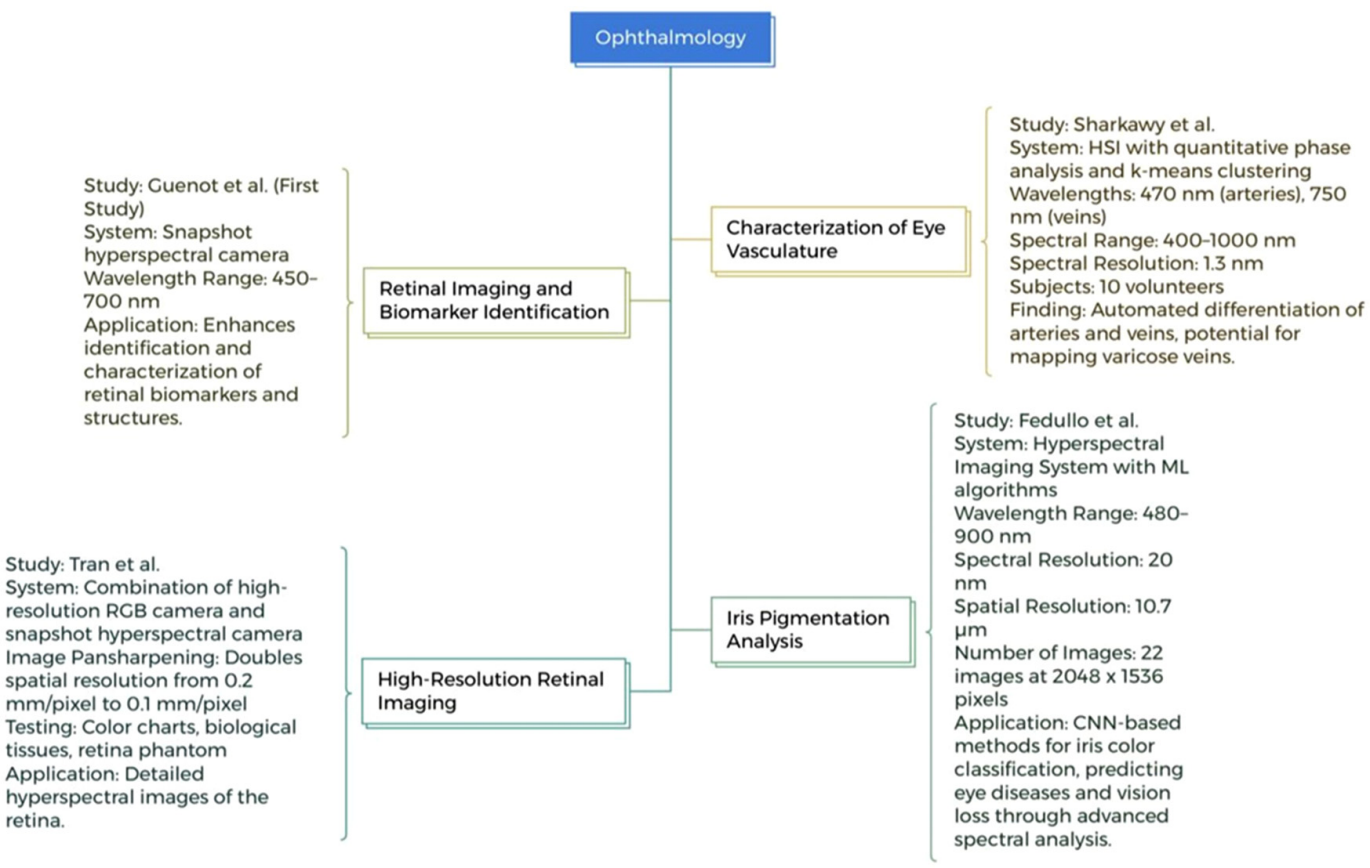
HSI applications in ophthalmology research.

A new snapshot HSI camera designed by Guenot *et al.* for retinal imaging enables exact spectral absorption measurement in the wavelength range of 450–700 nm with a resolution of 12 nm. This serves as a crucial tool for clinical and therapeutic research and advances the study of retinal biomarkers.^87^ Feddullo *et al.*, explored the incorporation of ML algorithms with the HSI to analyze the iris pigmentation. The system captured 22 images operating within a wavelength range of 480 to 900 nm, with a spectral resolution of 20 nm and spatial resolution of 10.7 µm. Using a CNN-based technique, the system effectively developed a classification framework of iris color. The study demonstrates HSI's potential in predicting eye diseases and vision loss through ML.^88^ An innovative hyperspectral eye fundus camera was employed by Selvander *et al.* to study the optical characteristics of choroidal nevi in the retina. The experiment consists of nine patients and the camera has a spectral resolution of 12 nm across the 450–700 nm range^89^ as illustrated in Fig. 5.

### G. Surgical operation

Vliet-Pérez *et al.* evaluated the efficacy of HSI in detecting ovarian cancer using surgically ejected tissue samples from 11 patients by employing the narrow spectral bandwidth (<15 nm) and in the NIR range 665–975 nm. On 26 samples, the ML algorithm was trained like an SVM classifier and achieved a classification power of 0.83, sensitivity of 0.81, and specificity of 0.70.^90^ The concept of applying HSI in brain tumor resection was investigated by Manni *et al.* through leveraging CNNs. To extract spectral and spatial information from *in vivo* specimens the 3D–2D hybrid CNN framework was developed with an operating optical range of 400–1000 nm and a resolution of 2–3 nm and showed overall accuracy of 80% in classifying tumors.^91^ A workflow for utilizing HSI was developed by Tran *et al.* to enhance nerve tissue differentiation in the course of surgery. The system implements SAM covering 400–700 nm with a high segmentation score (IoU) of 84.15% and 76.73% on the tissue samples of humans.^92^

**FIG. 6.**
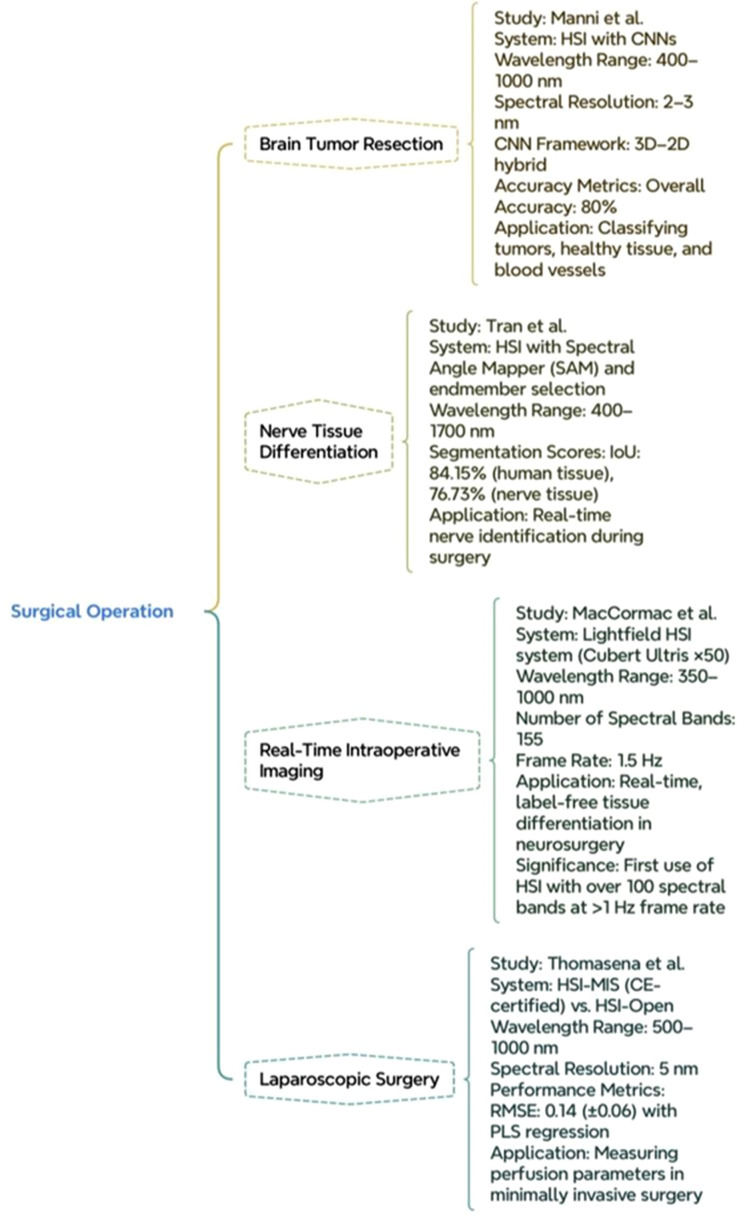
Enhancing surgical operations with HSI.

Macormac *et al.*, developed a light field HSI system designed for intraoperative neurosurgery applications by integrating Cubert Ultris ×50 camera with 155 spectral bands ranging from 350–1000 nm. This system exhibits the ability to capture hyperspectral data with less interference in surgical workflow.^93^ A study conducted by Thomasena *et al.*, adapted intraoperative HSI for laparoscopic procedures. This study involved 19 patients, measuring perfusion parameters within a spectral range of 500–1000 nm at 5 nm resolution. This approach enhanced accuracy with a root mean square error of 0.14 (±0.06) as seen in Fig. 6.^94^

### H. Wound assessment

In the burn wound assessment Promny *et al.*, emphasized the HSI by measuring perfusion in the NIR spectrum, with a spectral range of 500–1000 nm and a resolution of 5 nm. The examination involved 97 burns with different severities associated with the upper extremities. Deep learning algorithms like CNN are used to develop an accuracy of 87%. The total body surface area of the burns ranged from 1 to 56.5%, averaging 10.63%.^95^ For enhancing burn wounds Rex *et al.*, employed HSI to measure the volume fraction and oxygenation in depth to six skin layers. This system was performed in the bandwidth of 500 to 1000 nm along a spectral resolution of 5 nm by analyzing depth profiles of 43 patients and aided with a fast and reliable burn classification.^96^ Schulz *et al.*, evaluated an optical method for assessing burn depth using HSI. The study consisted of patients with 2nd and 3rd-degree burns. Operating in the VNIR spectrum from 500–1000 nm, the study predicted skin graft requirements within 72 hours with 92% accuracy, 92% sensitivity, and 71% specificity.^97^

**FIG. 7.**
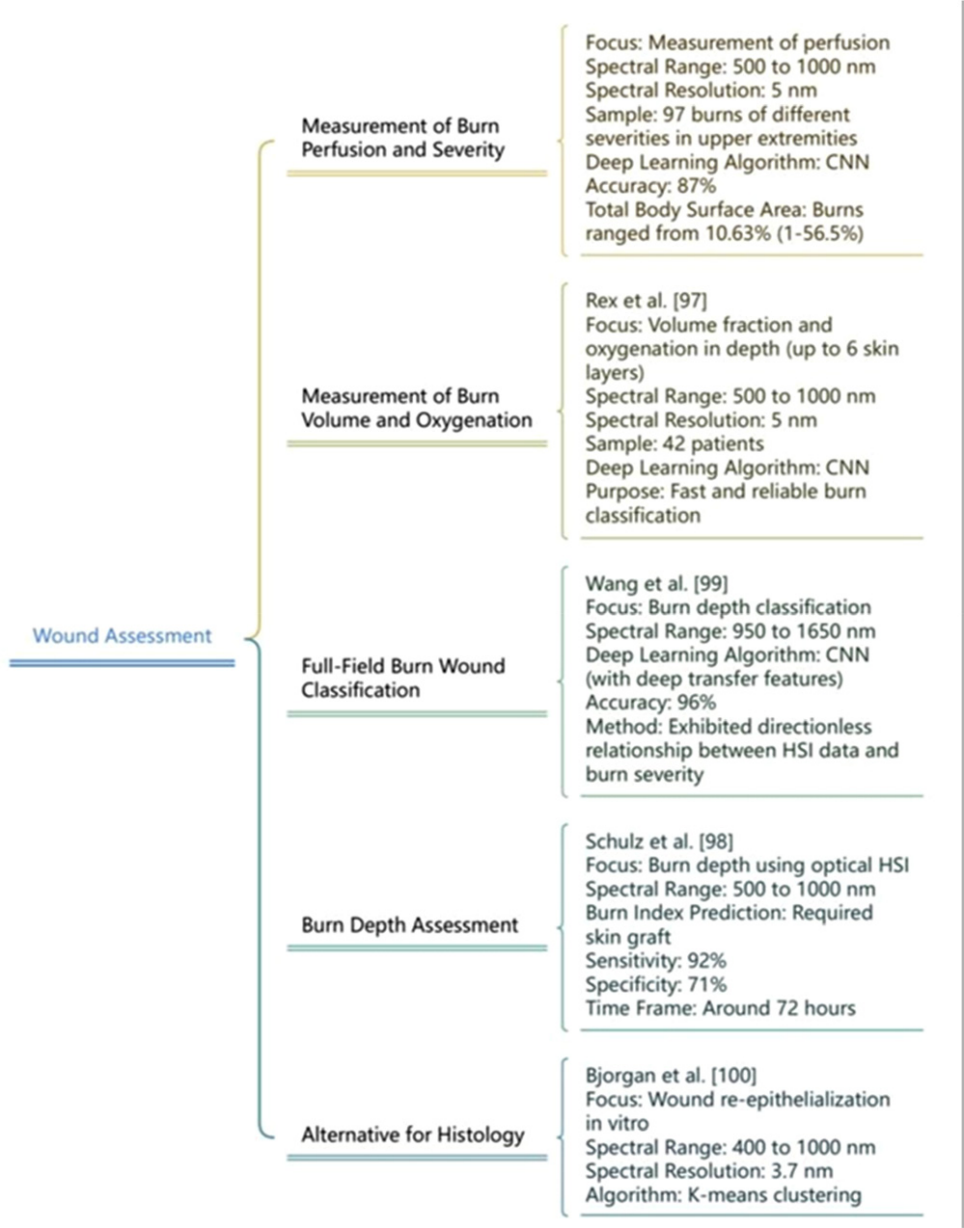
HSI applications in wound assessment.

An entire-field burn wound classification system was presented by Wang *et al.*, using NIR hyperspectral imaging, enhanced with deep transfer learning features. The system operated in the wavelength range of 950 nm to 1650 nm and CNN was employed to exhibit a directionless relationship between HSI data and burn severity, achieving 96% accuracy in burn depth classification.^98^ As an alternative for histology Bjorgan *et al.*, used HSI to examine wound re-epithelialization *in vitro*, This process spanned wavelength from 400–1000 nm with a spectral definition of 3.7 nm by applying k-means clustering for analysis, as illustrated Fig. 7 below.^99^

## IV. ADDRESSING TECHNICAL AND PRACTICAL CHALLENGES

**FIG. 8.**
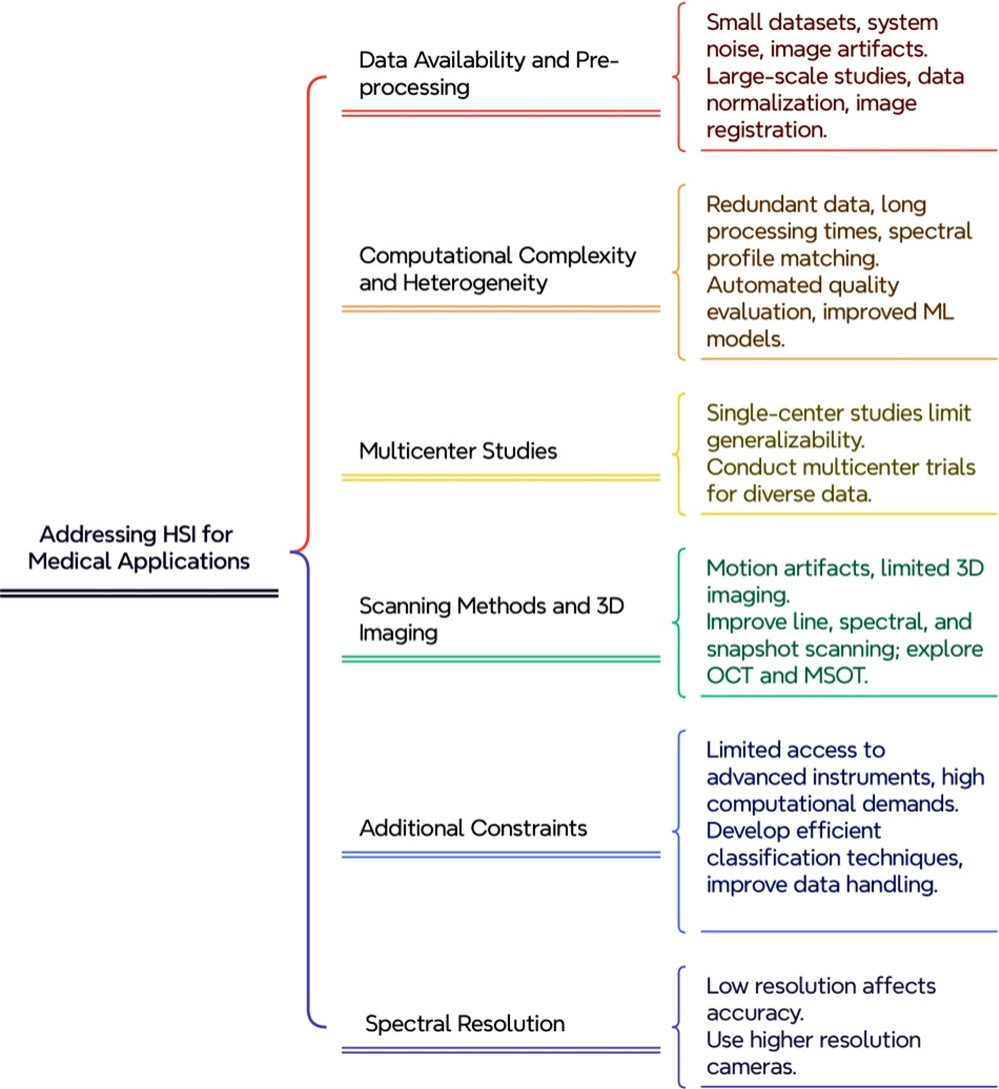
Technical and practical challenges in HSI for enhanced medical applications.

HSI offers a distinctive advantage over conventional imaging methods, like RGB, MRI, and CT, by capturing comprehensive spectral data, enabling detailed biochemical and structural analyses at the molecular level. This often results in higher diagnostic accuracy for early detection of diseases such as skin cancer and diabetic retinopathy. However, HSI faces integration challenges in clinical settings due to its complex data acquisition process, specialized training requirements, and significant computational needs for high-dimensional data storage and processing. This study highlights the potential of ML and data standardization to enhance HSI's clinical adoption by simplifying data interpretation and ensuring consistency across different HSI systems. With advancements in ML, HSI is expected to become more accessible and practical for routine diagnostics, complementing traditional imaging methods with its noninvasive, precise diagnostic capabilities and potentially surpassing them in specialized applications. To advance the medical application of HSI, it is crucial to address the existing challenges impacting its performance. This section emphasizes such key limitations that restrict the seamless integration of HSI in clinical workflows, as illustrated in Fig. 8.

### A. Data availability and pre-processing

Small size of the dataset certainly impacts the efficacy and validation of HSI in terms of medical application, which may lead to technical challenges. As discussed in the study by Wang *et al.*, the limited amount of dataset size 126 retinal images was considered to be a limitation on utilizing HSI for DR detection. Similarly, neurodegenerative research related to Parkinson's disease by Ueda *et al.*, is incapable of performing model validation with several classifiers, leading to potential overfitting due to the small data size. Large-scale studies are essential to address these limitations and ensure robust outcomes. In Hart *et al.*, study on chronic venous disease there is a relatively small number of limbs in each clinical class category further emphasizing the need for larger cohorts to evaluate preliminary findings. Other technique limitations include system noise and image artifacts caused by uneven illumination or redundant details of HSI sub-bands. To address these challenges, data normalization is important for converting hyperspectral radiance into reflectance or absorbance values that accurately represent biological properties. Developed and advanced preprocessing techniques like image registration, feature extraction, and classification algorithms help in extracting and classifying relevant spectral information. Particularly, Gaussian filters can reduce noise and enhance spectral fingerprint clarity^100^ Study of minor sample size and endpoint of heterogeneity which cause further complexity and potential inconsistency. The existing technology only allows static images, and confines real-time study of perfusion and continuous monitoring. In addition to clinical data, the study of cost efficiency is mandatory for long-term practicability.^101^

### B. Computational complexity and heterogeneity

The data generated from HSI are vast and often relayed with redundancy, which creates computational challenges. An automated quality evaluation system is rarely used due to the lengthy process involved in image acquisition and analysis, the key issue lies in an indirect method, that requires standardized calibration and model transfer procedure.^102^ A primary limitation of HSI is that doctors cannot instantly evaluate the hypercube. According to the research on automatic recognition of colon and esophagogastric cancer using ML and HSI by Collins *et al.,* ML is essential for learning and automatically detecting and reporting pertinent spatio-spectral patterns. A study by Wang *et al.* stated that although CNN-based models perform well, they have limitations in terms of simulating correlations and similarity between spectra. Likewise, there is a lack of models that work efficiently for classifying multimodal data. The main limitation of Parasca *et al.*, ’s study on HSI with ML for *in vivo* skin cancer margins assessment is the heterogeneity in histological subtypes. Eliminating ambiguities becomes more challenging while matching spectral profiles with biological samples, the inherent non-uniqueness makes this an extensive limitation of HSI. This limitation showcases the need for advanced algorithms.^103^

### C. Spectral resolution and range

The use of only one case and a small number of training pixels in the area directed only to the ulcer are considered to be limitations in a study that combines HSI with ML classifiers for diabetic leg ulcer assessment. A study of Urban *et al.* on tissue health diagnostics of the oral cavity revealed that the resolution of an HSI snapshot camera is the main constraint that makes it difficult to assess the structure of tiny capillaries. A camera with more resolution may be able to account for the motion artifact reduction that comes with a lower resolution. According to Sharkawy *et al.*, ’s study, automated hyperspectral imaging for non-invasive assessment of human eye vasculature, the main limitation to consider is the utilization of a specific wavelength range of 400–950 nm. This range may have missed certain significant spectral characteristics crucial for vascular mapping. In addition to spectral resolution, the spectral range of hyperspectral imaging (HSI) is a key factor that affects its effectiveness in medical diagnostics. While spectral resolution determines the ability to distinguish between closely spaced wavelengths, spectral range defines the extent of wavelengths captured, influencing the technique's capability to detect various biochemical and structural features across different tissue types. Expanding both the resolution and range could enhance the diagnostic power of HSI by capturing a broader and more detailed spectral profile, enabling improved differentiation of tissue properties. Addressing both aspects is essential for advancing HSI applications in clinical settings.

### D. Multicenter study

Single-centered studies are considered to be the main limitation for a massive amount of research studies that use HSI for medical applications, which are restricted by factors such as limited scientific rigor, lack of generalizability, and contradicting results. To address these limitations, there is a need for multicenter studies that take place at various medical institutions despite the ethnicity, race, and social constraints. It accomplishes broader patient diversity from the diverse patient population with various geographical locations to enhance diversity generalizability and studies across various demographics. By incorporating multicenter studies the total sample size is significantly increased, which improves the power of statistical analysis of the study and results in more accurate conclusions. Collaborating among various institutions allows the sharing of resources and expertise with technical methodologies for innovative approaches in terms of research. The results from the multicenter studies are often considered more credible by the regulatory bodies and scientific community. Thus, facilitating regulatory approval is more effective as it signifies the efficacy and safety of interventions across a wider population.

### E. Scanning methods and 3D imaging

Line scanning methods are contributed to produce high spatial and spectral resolution but they mostly struggle with highly motile targets considering their mechanical nature. On the other hand, the spectral scanning method serves to provide a stable image without mechanical movement but it has less spectral resolution. Despite this, it is more suitable in clinics for its simplicity and stability. The snapshot method captures both spatial and spectral information simultaneously but it requires improvements in terms of both resolution and range.^104^ In a clinical setting, performing quantitative 3-D HSI remains challenging, though it is assisted as crucial for examining lesion boundaries or diagnosing metastasis, the primary issue is measuring broadband light signals which will scramble 3D depth information while using the conventional HSI method and it is impractical to cut the tissues into thin slice for 3D imaging and for rapid acquisition. An alternative solution exists like spectral optical coherence tomography (OCT) and multispectral optoacoustic tomography (MSOT) to obtain 3D spectral information but these still need advancement in optical systems for practical clinical application.^105,106^

### F. Additional constraints encountered in HSI

Limited access to the powerful commercially available instrumentation, the plenty of data generated, for the requirement of analyzing complex data and algorithms, and the absence of academic training clogs extensive application are some of the other limitations of HSI.^107^ High computational complexity is also an unresolved challenge, and efficient classification techniques are necessary to handle hyperspectral data's vast dimensionality and complexity.^108^ In operating theaters or clinics, several limitations hinder the application of general HSI. The machinery's immense size and long-delayed imaging time are inclined to be significant issues inclusive of the high risk of artifacts from patients or instrument movements.^109^ High data redundancy arises from the strong correlation between adjacent bands and variability in hyperspectral signatures, the complex dimensionality of hyperspectral data, known as the invocation of dimensionality, complicates automatic analysis.^110^ Advanced methods for analyzing HSI in the medical field are not as developed or widely applied compared to those in remote sensing.^111^ The light absorption and scattering in tissues will limit the image depth which results in an optical penetration depth restriction that differs with tissue composition and wavelength. Even though NIR light penetrates deeper than visible light, it still attains millimeter-level depths in skin tissue. This limits *in vivo* tumor detection of exterior organs which are accessible through endoscopic methods and limits optical imaging modalities to slices or shallow surfaces.^112^

## V. STATE-OF-THE-ART HYPERSPECTRAL IMAGING SYSTEMS IN MEDICAL APPLICATIONS

**FIG. 9.**
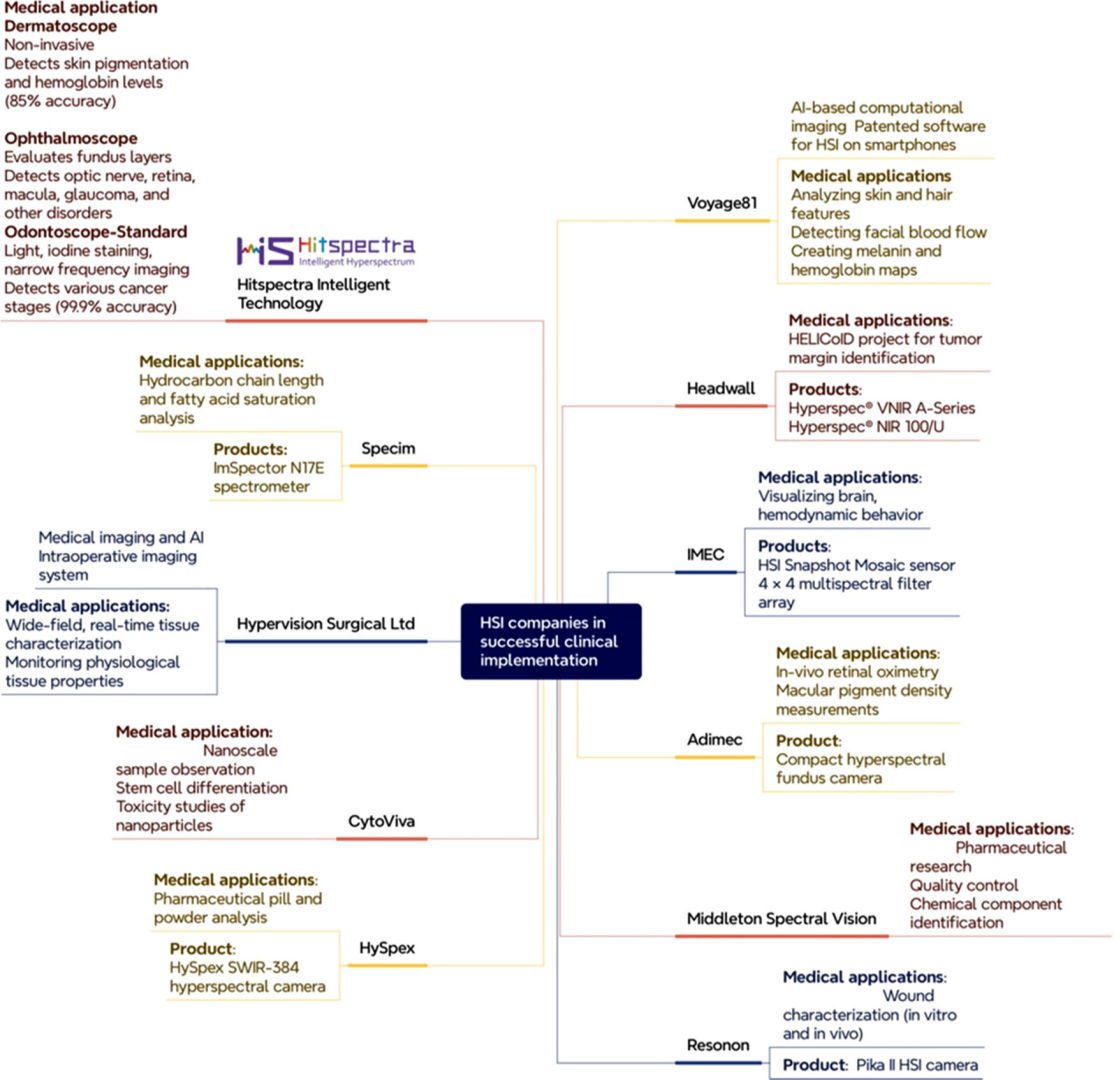
Leading companies in clinical HSI.

The following section highlights 11 companies that have been in medical practice and recruitment of HSI-based artifacts in clinical settings with successful implementation and results demonstrate the foresight and feasibility of integrating HSI into clinical workflows, as summarized in Fig. 9.

Hitspectra Intelligent Technology Co., Ltd. is a company that specializes in building advanced identification systems; it uses a noninvasive tool called a dermatoscope, which can detect aberrant skin pigmentation and hemoglobin levels with an accuracy of 85%. An ophthalmoscope allows for the evaluation of every layer of the fundus for diseases affecting the optic nerve, retina, macula, glaucoma, and other disorders. It is also employed in the odontoscope-standard with light, iodine staining, and narrow frequency imaging, with various waveforms corresponding to distinct cancer stages with an anticipated accuracy of 99.9%. Integrating HSI into odontoscopes enhances oral health diagnostics by detecting subtle color changes in oral cells for early diagnosis of oral cancer by providing physicians with a wide range of data to treat the disease more effectively.

Voyage81 is a deep-tech startup specializing in AI-based computational imaging. It stands out as the sole company globally to have developed patented software that enables HSI on smartphones using its advanced physics-based algorithms. Proprietary algorithms utilize the RGB data of each pixel as a color signature. With a pre-collected hyperspectral database, ML models match each color signature to an optimal hyperspectral signature, enabling pixel-level spectral recovery. Medical applications of this technology include using a basic smartphone photo to create maps of hemoglobin and melanin, detecting face blood flow, and analyzing skin and hair traits.

Headwall leverages advanced spectral technology to address complex challenges for both end users and OEMs. In the European collaborative project Hyperspectral Imaging Cancer Detection (HELICoID), during brain surgery, headwall's Hyperspec ^®^ imaging sensors are used to detect tumor margins in real-time. The HELICoID demonstrator's acquisition system includes two hyperspectral cameras supplied by Headwall Photonics: the Hyperspec^®^ VNIR A-Series and the Hyperspec^®^ NIR 100/U.^113^

Interuniversity Microelectronics Centre (IMEC) has a long tradition of success in the sector of HSI prototyping and research. The IMEC camera is employed to visualize brain hemodynamic behavior, thereby improving the understanding of cerebral metabolism and neurovascular coupling. This imaging system incorporates a 4 × 4 multispectral filter array built on a CMOSIS-CMV2000 CMOS chip that powers an HSI snapshot mosaic sensor.^114^

Adimec's Gentific cameras are utilized for imaging systems in clinical diagnostics in hospitals and laboratories to maintain standards for medical imaging tools and to enhance diagnostic performance. They use compact hyperspectral fundus cameras for assessing macular pigment density and retinal oximetry *in vivo*. The detector chip is integrated into 80 mm^2^, 400 g Adimec quartz series camera body (Q-2A340).^115^

Middleton Spectral Vision is a company specializing an advanced HSI systems and spectroscopy. This organization uses HSI to recognize and quantify samples of chemical components with the bands of NIR and SWIP for pharmaceutical research and quality control management. They enhance precision and efficacy of manufacture by serving with blending, tracking tablet manufacture, and identifying counterfeit products.

Resonon develops and provides hardware and software for HSI across laboratory, industrial, and airborne settings. NIST and the Ohio State University researchers utilized a Resonon Pika II HSI camera, the predecessor to the Pika L, for wound characterization. Hyperspectral data cubes from *in vitro* and *in vivo* models were acquired by operating the PIKA II hyperspectral line scan imager.^116^

HySpex is recognized as a leading brand in both airborne and ground-based HSI, known for the stability, flexibility, and high-quality data of its sensors. A hyperspectral HySpex SWIR-384 camera was utilized to determine the concentration and homogeneity of chemical components in pharmaceutical pills and powders. Operating at a 930–2500 nm spectral range using a spectral sampling of 5.5 nm, the camera features 384 spatial pixels and achieves a spatial resolution of 52 µm. It captures images with most transport and sorting systems at reliable speeds, allowing for real-time analysis.

CytoViva custom designs HSI solutions for micro- and macro-scale research. They possess an Enhanced Darkfield Microscope illuminator with the CytoViva HSI system, allowing label-free, non-invasive cellular change images like oil droplet formation during adipogenesis and calcium deposition during osteogenesis.^117^ CytoViva's HSI is used to investigate the toxicity mechanisms of silver nanoparticles (AgNPs) and silver ions (Ag+) on Vero 76 cells. In this study, HSI provided label-free, noninvasive imaging to analyze the effects of AgNPs and Ag+ on cellular components.^118^

Hypervision Surgical Ltd is a company providing the future of surgery using AI-powered hyperspectral imaging. Their approach is non-contact, non-invasive, using quantitative AI imaging on physiological tissue properties, capable of real-time AI-driven, wide-field tissue classification to a diagnosis of tumor, normal tissue, nerves, and blood vessels.

Specim is a successful globally recognized supplier of HSI systems and solutions, it provides a wide range of cameras and wavelength accessories from visible to thermal. One of its notable products is the line-scanning NIR-HSI system equipped with the ImSpector N17E spectrometer. This system has been utilized in near-infrared HSI applications, such as analyzing hydrocarbon chain length and fatty acid saturations in mouse livers by integrating ML algorithms.^119^

## VI. FUTURE DIRECTION AND RECOMMENDATIONS

To summarize, despite HSI having exhibited tremendous potential for medical applications, further research and investment are required to address the remaining limitations, barriers, and challenges that hinder their clinical implementation. Future research should concentrate on optimizing algorithms, creating technical guidelines, and conducting multicenter trials and research to validate the clinical effects of HSI. Strong regulatory frameworks and interdisciplinary collaboration are essential for promoting the adoption of HSI in clinical practice.

Multidisciplinary cooperation and financial initiatives are essential for rapid invention of HSI technology in medical settings. A distinct regulatory framework combining patient-oriented design values and ML standard practices is required for AI/ML-based software as a medical device to ensure user clarity. Regulatory bodies that involve the FDA and CE must develop and foster regulatory science techniques concerning algorithm robustness, bias, and real-world performance, this collaborative motive will make it more accessible for HSI-based medical devices to be authorized and performed in various sectors, promoting public safety, effectiveness, reliability, and quality.

In this paper, HSI is demonstrated to be a vital instrument for the early detection and diagnosis of a wide range of medical conditions, including areas such as wound assessment, surgical operation, ophthalmology, cancer, dermatology, periodontal diseases, neurodegenerative diseases, and diabetes. These studies have concluded that HSI can achieve impressive results, especially in regard to early stage disease detection. To detect dysplasia via image-enhanced endoscopy, the American Society for Gastrointestinal Endoscopy (ASGE) has implemented the preservation and incorporation of valuable endoscopic innovation (PIVI), which requires a minimum performance threshold of 90% sensitivity and 80% specificity per patient.

There has been a growing amount of interest among academics, medical, and various regulatory bodies for developing AI/ML-based medical devices.^120^ The Food, Drug, and Cosmetic Act (FDA) published guidelines for AI/ML-based medical devices in January 2021.^121^ Under the USA's FDA Act, Switzerland's Therapeutic Product Act, and the European Union (EU) Council Directive 93/42/EEC, the AI/ML-based software for the detection, prevention, and treatment of health issues is regarded as a medical device.^122^ Due to the high risk associated with medical devices, the FDA regularity commission offers three methods by which it approves medical devices, the first pathway is premarket approval which is designed for high-risk devices, the second is a de-nova premarket review for low or moderate-risk devices and final is 510(k) pathway.^123^ Unlike the USA, European nations do not regulate medical devices: in the case of low-risk medical devices, the manufacturers hold the responsibility to ensure that products are in risk class 1 and comply with regulations without violating any approval process. AI/ML-based medical devices have been attracting increasing interest and demand firm regulatory oversight.

The spectrometer often struggles to detect low concentrations of biomarkers, leading to an increased risk of false positives. Some require extensive preparation of samples, which can be time-consuming with complex data interpretation required for accurate and precise results. It does not provide high spectral resolution with a limited spectral range. To address this limitation, Spectrum-Aided Vision Enhancer (SAVE) algorithm is performed, which can covert WLI and NBI without the requirement of spectrometer to enhance endoscopic diagnosis by transforming WLI to a significant wavelength, which improves contrast for better tissue and abnormality detection. It converts RGB images to HSI images by employing a color calibration process with a Macbeth color checker by converting images from the standard RGB to CIE 1931 XYZ color space. The method emphasizes accurate color representation and clear image quality, which enables more accurate medical diagnoses by overcoming HSI restrictions.^124^

To effectively integrate ML and DL with HSI in healthcare, several advancements are recommended. First, specialized algorithms tailored to HSI should prioritize interpretability, ensuring insights are accessible for healthcare providers without ML expertise. Next, dataset standardization and augmentation are essential to create diverse, representative datasets that improve model generalizability and reduce diagnostic biases. Additionally, robust regulatory frameworks are necessary to ensure ML/DL diagnostic tools meet safety and efficacy standards, with guidance from regulatory bodies like the FDA and CE facilitating smoother clinical adoption. Interdisciplinary collaboration between developers, healthcare professionals, and regulatory entities is also crucial for aligning technological solutions with clinical needs, enhancing workflow integration. These combined efforts aim to make ML/DL-based HSI systems more reliable, efficient, and user-friendly, enabling noninvasive, precise diagnostics that improve patient outcomes and support clinical decision-making.

The deep recurrent convolution neural network (DnRCNN) model recruits a selective recurrent memory unit (SRMU) to capture interband and inner band correlative features. This unit is subjected to extracting correlated features in spectral and spatial domains to express the spectrum and spatial information loss. To eliminate striping artifacts and sustain scene details, the DnRCNN model integrates features from the SRMU combined with a novel recurrent fusion (RF) strategy. To address the scene adaption challenges this method helps in striking a balance between noise reduction and detail retention. The model effectively removes spectral distortion in textural regions and prevents over-smooth effects by emphasizing extended correlation features to retain spectral and spatial contents together. The simplified gated mechanism is employed to reduce the model size and accelerate convergence, tackling the challenge of parameter adjustment inherent in low-rank-based methods.^125^ A study by Winkler *et al.*, used CNN, which recently gained regulatory approval as a medical device for the European market (Moleanalyzer-Pro; FotoFinder Systems GmbH, Bad Birnbach, Germany), to diagnose melanoma through dermoscopic image analysis the system achieved results of high sensitivity and specificity, particularly sensitivities exceeding 93% for melanoma detection.^126^

Khodr *et al.*, investigated a review of dimensionality reduction techniques aimed to address both linear and non-linear dimensionality mitigation techniques which direct the challenges of high dimensional data.^127^ An ML method dimensionality reduction swaps out the high-dimensional data with lower-dimensional data, thus the dimensionality of the inputted data can be reduced and prediction accuracy increased.^128^ Dimensionality reduction is a pre-processing method in HSI classification that eliminates irrelevant redundant, noisy data to increase learning accuracy and reduce training time. Any sort of high-dimensional data can be evaluated using dimensionality reduction algorithms particularly crucial for data analysis associated with remote sensing.^129^ The common dimensionality reduction methods utilized by HSI include PCA, uniform manifold approximation and projection (UMAP), and self-organized maps (SOMs).^130^ PCA is a technique that reduces the spectral dimensionality of hyperspectral data cubes in clinical settings and applications. The foundation of PCA relies on the observation of neighboring bands in HSI that are strongly correlated and share similar object-related information; consequently, the PCA transforms the original data to remove these correlations.^131^ As an input end member, it can also pick the best wavelength. For medical hyperspectral datasets, nonsupervised PCA is the most used dimensionality reduction technique.^132^ UMAP is a technique for reducing dimensionality nonlinear within data that achieves a balance between global and local structure. It works by calculating the range between points in high-dimensional and low-dimensional space. UMAP uses stochastic gradient descent to decrease the difference between high- and low-dimensional distance. UMAP is a nonlinear dimensionality reduction technique that achieves a balance between global and local structures in data. It works by calculating the range between points in high-dimensional and low-dimensional space. This technique is notable for its rapid execution, high repeatability, and flexibility in analyzing high-dimensional data.^133^ SOM is an effective and powerful software tool that is utilized for visualizing multidimensional data.

In terms of medical imaging databases, it is crucial to have access to open-source databases for advanced research and discovering various models for detecting and diagnosing disease through ML models. The International Skin Imaging Collaboration (ISIC) data are a collection of scanned images of skin, metadata, and expert annotations used by ML researchers to examine medical images. These databases are a vital tool for cancer research and are used in several types of studies. The availability of open-source databases similar to ISIC is required to eliminate significant limitations like data availability, the researcher from worldwide gets access to comprehensive datasets which would facilitate advancement in medical research and treatment methodologies across various diseases between high-dimensional data into simple geometric relationships on a low-dimensional display.

## VII. CONCLUSION

Despite HSI being denoted as an advancement tool, it is important to feature its limitations for wide clinical adoption. Future research investments are required to overcome current limitations and upgrade clinical implementation by focusing efforts on systemizing optimal algorithms, establishing technical guidelines, implementing strong regulatory frameworks, and potential collaboration between researchers. Healthcare professionals will benefit from incorporating HSI in patient care. Strict regulations are required for AI/MI-based medical diagnostic devices to ensure safety and effectiveness. Regulatory bodies such as the FDA and CE provide essential guidance in the approval process. HSI can prevail as an important tool in modern medical diagnosis by defeating the existing barriers. It can be presented as a non-invasive tool with an accurate and timely diagnosis that ultimately improves patient outcomes.

## Data Availability

The data that support the findings of this study are available from the corresponding author upon reasonable request.
